# Dexamethasone implant in silicone oil: in vitro behavior

**DOI:** 10.1186/s40942-018-0127-x

**Published:** 2018-06-20

**Authors:** Erick Omar Flores-Villalobos, J. Abel Ramírez-Estudillo, Atzin Robles-Contreras, Jacqueline L. Oliva-Ramírez

**Affiliations:** 1 Retina Department, Hospital “Nuestra Señora de la Luz” P.A.I., Ezequiel Montes 135, Tabacalera, Cuauhtémoc, 06030 Mexico City, Mexico; 2 Biomedical Investigation Center, Hospital “Nuestra Señora de la Luz” P.A.I., Ezequiel Montes 135, Tabacalera, Cuauhtémoc, 06030 Mexico City, Mexico

**Keywords:** Dexamethasone, Silicone oil, Tamponade

## Abstract

**Background:**

To determine the effect of the silicone on the dexamethasone intravitreal implant.

**Methods:**

Basic, experimental, prospective and transversal study performed at the hospital “Nuestra Señora de la Luz” in Mexico City. One dexamethasone implant was placed in a test tube with 4 mL of each tamponade medium: 1000cS, 5000cS and heavy silicone oil; basic saline solution was used as the control medium. Photographs were taken weekly for 12 months. 200 µL samples were taken from each medium at 24 h, 1, 2 weeks and monthly for 12 months. ELISA test was performed to quantify dexamethasone release in every sample. An inflammatory stimulus was created and later exposed it to every sample in order to test their anti-inflammatory capacity by cytokine analysis using cytometric bead array. Statistically significant results were obtained with p < 0.05.

**Results:**

Photographic follow-up showed disintegration of the implant in control medium. Implants in silicone oil suffered no changes during follow-up. Dexamethasone levels in control medium showed stability from month 2 to 12. Silicone oil mediums showed irregular dexamethasone release during the 1 year period. Dexamethasone in control medium had inhibitory effects on TNF-α starting at 24 h (p < 0.001) and remained stable. Dexamethasone in 1000cS silicone oil showed inhibitory effects from month 2 (p < 0.001) until month 6 (p < 0.001). Implants in denser silicone oils showed no inhibitory effects in any of the samples.

**Conclusions:**

Denser mediums altered the implant pharmacokinetics and showed no anti-inflammatory effects even when concentrations were quantified at levels similar to control medium in vitro.

## Background

Proliferative vitreoretinopathy (PVR) is the most common cause of complicated retinal detachment, mainly caused by an inflammatory and proliferative cellular reaction that challenges the conventional treatment. In the modern era of vitreoretinal surgery, silicone oil (SO) is the most used internal tamponade medium to treat complicated retinal detachments [1–5]. Clinical usage of SO in treating retinal detachment was first introduced in 1960s even before the introduction of pars plana vitrectomy [6] and by the late 1980s it established its role as an internal tamponade by achieving higher anatomic success, especially in cases of PVR that were previously thought untreatable [7].

Silicone is made up of repeating units of siloxane, which consists of silicone and an oxygen molecule, with the chemical formula (–Si–O–). Heavier-than-water SO is a solution of a mixture of polymethylsiloxane and semifluorinated alkanes or alkenes, and a methyl or trifluoropropyl side chain can be added to the siloxane unit to form polytrifluoropropylmethylsiloxane, also known as fluorosilicone oils. Lighter-than-water SO (conventional SOs) consists of polydimethylsiloxane (PDMS), which is a mixture of siloxane with two attached methyl side chains and vary with regards to their viscosities, measured in centistokes (cS). PDMS has a specific gravity of 0.97 at 25 °C regardless of their viscosity (1000cS SO, 2000cS SO or 5000cS SO), which is lighter than water and fluorosilicone oils have a specific gravity of 1.25–1.3, which is heavier than water [8]. Heavy SOs represents an evolution of the fluorinated SOs developed in the 1980s. Of all the commercially available heavy SOs, Densiron 68 (D-68), a solution of 70% 5000 cS SO and 30% F_6_H_8_ with an specific gravity of 1.06 and a viscosity of 1350 cS, is the most commonly used worldwide and the clinical experience with this agent is extensively reported [9–13]. Retinal detachments with inferior breaks or complicated by posterior PVR is the most debatable scenario comparing 1000cS SO and 5000cS SO versus D-68 internal tamponades. In this cases, evidence has failed to show any significant difference using heavy SO over conventional SO regarding anatomic and functional success [14].

These vitrectomized, silicone oil-filled eyes are frequently accompanied with other retinal manifestations, due to the chronic, inflammatory and proliferative nature of the underlying diseases [15–17]. Growing experience with the use of new drug delivery systems [18–23] such as the intravitreal dexamethasone implant has proven to be effective as an anti-inflammatory option for vision improvement and reducing the risk of vision loss in several multicenter clinical assays [24–29].

However, even when the vitreoretinal pharmacokinetics of the intravitreal dexamethasone implant had been reported to be similar between vitrectomized and nonvitrectomized rabbit eyes, according to Chang-Lin et al. [30]; the information available on the behavior of the dexamethasone intravitreal implant in vitrectomized silicone oil-filled eyes is limited to some case reports where the implant remained encapsulated, trapped against the retina or behind the iris with no further analysis [31–33].

Based on the hypothesis that the dexamethasone release will decrease when the implant is placed in silicone oil, we designed this study with the goal of determining if this modifies its in vitro concentration and/or therapeutic effect.

## Methods

### Dexamethasone implant in different silicone oils

The 0.7 mg dexamethasone implant was placed in a 5 mL test tube filled with 4 mL of each tamponade medium: SO-1000cS, SO-5000cS, D-68 (heavy silicone oil) and BSS (basic saline solution—as control). One DEX implant was injected for each one of the test tubes.

**Fig. 1:**
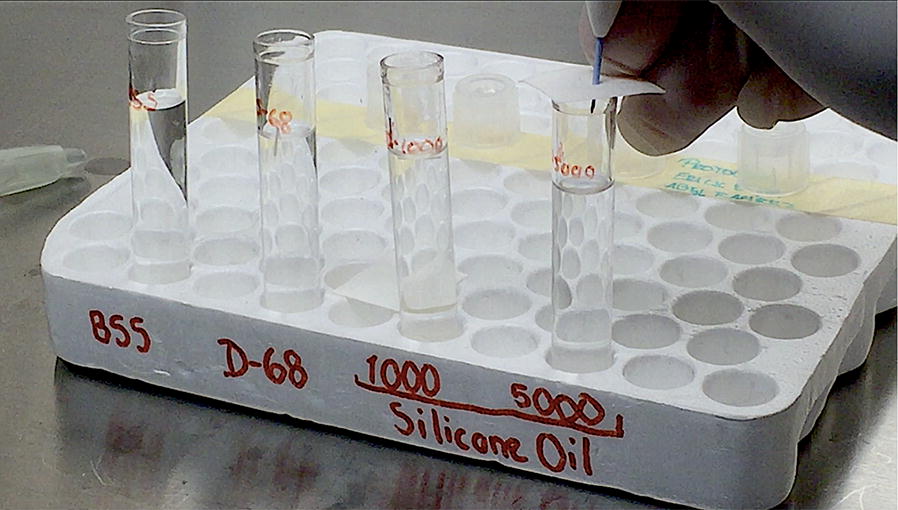
Dexamethasone implant placement inside the test tubes. Image shows the implant injection inside the 5000cS silicone oil-filled test tube through a sterile filter paper. One 0.7 mg dexamethasone implant was injected in every test tube previously filled with 4 mL of each tamponade medium, 1 mm thick sterile filter paper was placed on top of the tubes to simulate the scleral wall stiffness in the human eye. *BSS* basic saline solution, *D-68* heavy silicone oil, *1000* 1000cS silicone oil, *5000* 5000cS silicone oil

The implant placement was performed by the same retina surgeon performing the experiment using the original applicator, placed at 90º over a 1 mm thick sterile filter paper marked at the center to simulate the scleral wall surface in a human eye (Fig. 1).

After the implant injection, the test tubes were placed in a rack and stored inside an incubator at 37 °C during the twelve months follow up and were only taken out to a flow hood for sample acquisition.

### Sampling

Two hundred microliters (200 µL) samples were taken from each medium with a 200 µL yellow pipette tip at the center of the total volume of the test tube, adjusting the distance as the volume in the test tube decreased after every sample. Samples were acquired at 24 h, 1, 2 weeks and monthly for 12 months. Sixty samples were stored at − 80 °C until analysis.

### Photographic monitoring

Color photographs were taken weekly for 12 months since the implant placement in each medium, monitoring the migration, position and disintegration of the implant in each medium.

### Dexamethasone quantification

The samples obtained from the different mediums where the implant was placed were defrosted at room temperature. ELISA test (Neogen corporation, Lansing, Michigan, USA) was performed to quantify dexamethasone following the manufacturer instructions.

### Isolation of mononuclear cells

Using heparinized diluted peripheral blood 1:2 relation (vol/vol) in phosphate buffer saline (PBS) with a PH of 7.4, peripheral mononuclear blood cells (PMNBC) were separated by density gradient with Ficoll and spin-dried at 1800 rpm for 30 min at room temperature. Posterior to spin-dry, cells were collected with a Pasteur pipette in the leukocyte ring and quantified by exclusion with trypan blue in a Neubauer chamber. 1 × 10^5^ cells were placed in each well. Later, RPMI 1640 non-supplemented medium was added for 24 h.

### Mononuclear cell stimulation

After 24 h with RPMI 1640 non-supplemented medium, it was replaced with RPMI 1640 supplemented with bovine fetal serum at 10%. After this, every well with 5 µL of each sample was pre-incubated to later incubate them with lipopolysaccharide for 24 h. Supernatants were collected after this time and stored at − 80 °C.

Non-stimulated cells were used as a control medium. As a positive stimulation control, cells stimulated only with lipopolysaccharide (LPS) were used. The inhibition control was made using DEX + LPS.

### Cytokine analysis

Supernatants obtained in the cell stimulation were processed by cytometric bead array technology (CBA, Human inflammatory kit-BD biosciences, CA, USA) the cytokines included in the kit were: Interleukine-1b (IL-1b), IL-6, IL-8, IL-10, IL-12 and TNF-α. The supernatants (50 μL) from each well were incubated with the beads for 3 h, and the beads were washed away and then recovered following manufacturer specifications (BD biosciences, San Diego, CA, USA). Concluding incubation time, samples were acquired in a flow cytometer FACS CANTO II. Analysis of the results was made with FACS DIVA software. TNF-α levels were chosen over the other cytokines included in the kit and the samples were considered as positive if the produced results were above the detection limit (TNF-α: 3.8 pg/mL).

### Statistical analysis

Results were analyzed with descriptive statistics and later subject to analysis of normality. Comparisons between different groups of study were made with a repeated measures ANOVA test using GraphPad Prism software v5.0. Statistically significant results were considered with a value of p < 0.05.

## Results

### Photographic follow-up

**Fig. 2:**
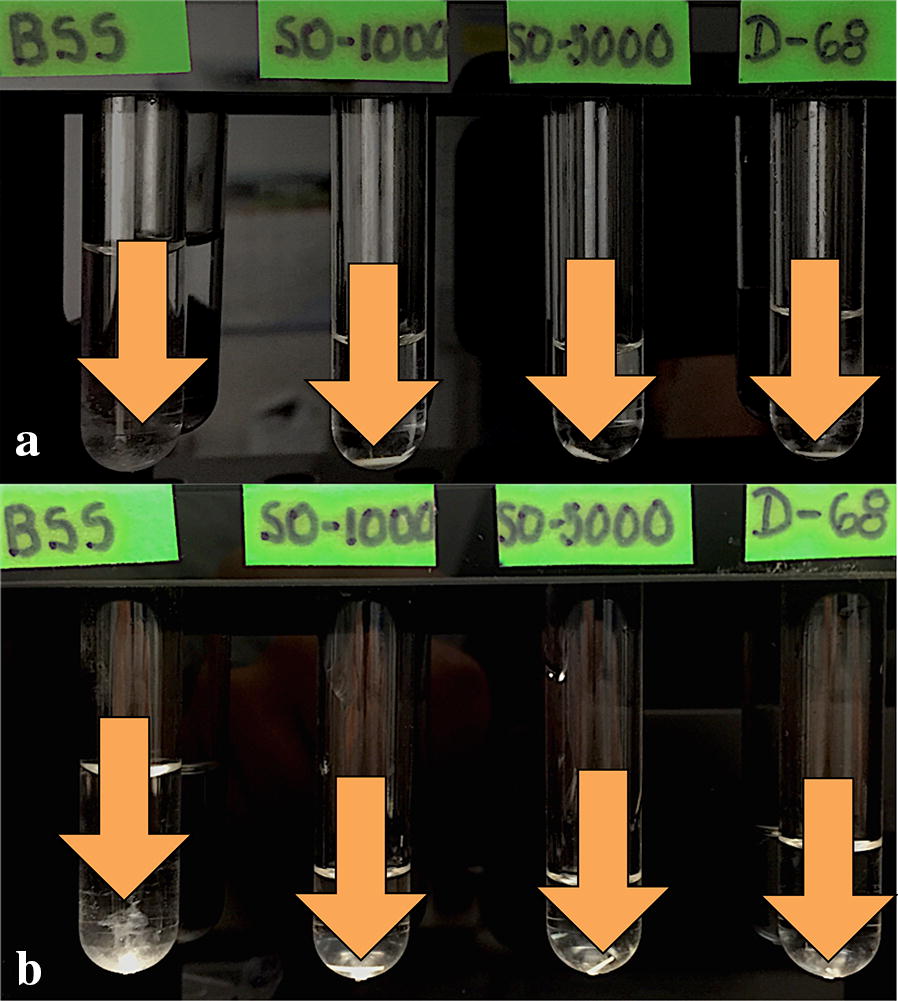
**a** Color photograph taken at month 9. Image shows the implant status in the test tubes at month 9. First arrow from left to right: partial disintegration of the implant in BSS surrounded by a turbid appearance corresponding to released particles of the polymer, approximately one-third of the implant still preserves its original cylindrical shape. From left to right: second, third and fourth arrows point the implants placed in 1000cS, 5000cS and heavy silicone oils. The implants remain at the bottom of the tube and no macroscopic signs of disintegration can be observed. **b** Color photograph taken at month 12. Image shows the implant status in the test tubes at month 12. First arrow from left to right: complete disintegration of the implant in BSS, scattered particles suspended at the bottom of the tube with no visualization of the implant. From left to right: second, third and fourth arrows point the implants placed in 1000cS, 5000cS and heavy silicone oils. The implants remain at the bottom of the tube and no macroscopic signs of disintegration can be observed

In the photographic follow-up we observed that the implant had no modifications in the BSS control medium until month 9 (Fig. 2a), after this it began to disintegrate until complete dissolution in the control medium at 12 months (Fig. 2b). As for the different silicone oil mediums, we observed no physical changes during the 1-year follow-up, and the implants remained at the bottom of each test tube.

### Dexamethasone quantification

**Fig. 3:**
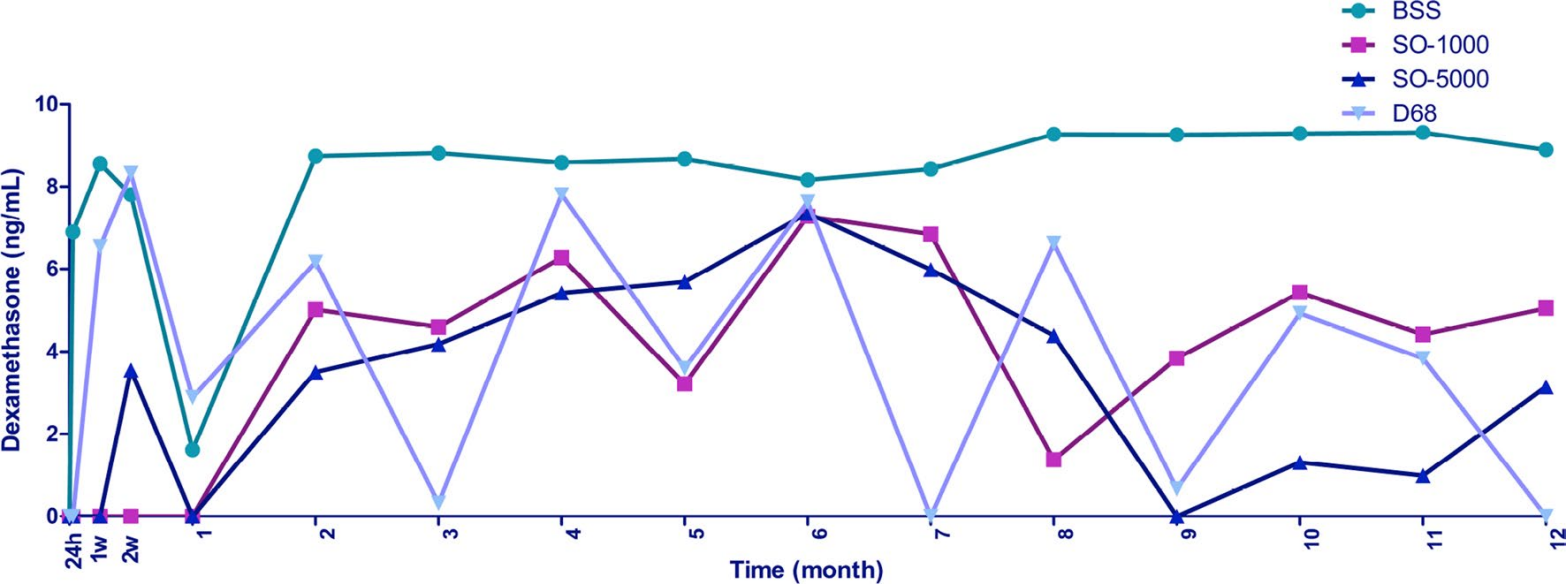
Quantification of dexamethasone released from the implants in each sample at different times. One 0.7 mg dexamethasone implant was injected in a test tube filled with 4 ml of different mediums: BSS, SO-1000, SO-5000 and D-68. Samples were obtained at different times: 24 h, 1, 2 weeks and monthly for 12 months. ELISA test was performed to quantify the released drug and reported in ng/mL. *BSS* basic saline solution, *SO-1000* 1000cS silicone oil, *SO-5000* 5000cS silicone oil, *D-68* heavy silicone oil, *h* hours, *w* weeks

Figure 3 shows DEX levels in each medium during the 12-month period. In the case of the control medium (BSS), levels of DEX showed stability from month 2 to 12, keeping an average concentration of 8.85. ± 0.39 ng/mL.

As for the silicone oil mediums, an irregular behavior was shown caused by a fluctuation in the DEX concentrations since the 24 h samples. Considering that stability of the DEX concentrations was reached since month 2 based on the behavior of the control medium (BSS), silicone oil mediums showed average DEX concentrations from the second month of: SO-1000cS: 4.84 ± 1.68 ng/mL; SO-5000: 3.81 ± 2.30 ng/mL; D-68: 3.77 ± 3.10 ng/mL.

We observed detectable levels of the drug in every sample from control medium with a release peak at 24 h, followed by a significant drop at month 1 and stabilization with sustained release from month 2 to 12; DEX released from the implants in silicone oil showed an irregular fluctuation of the drug levels during the 1-year follow-up. SO-1000cS showed quantifiable levels of the drug starting at month 2 with a marked downward fluctuation at month 5 and month 8 to finally reach the initially detected levels. SO-5000cs showed quantifiable levels of DEX at 2 weeks followed by a drop to absolute zero levels to a later upward trend to reach peak concentrations at month 6, after this point, levels of DEX showed a monthly drop to zero levels at month 9, to finally reach the initially detected levels. D-68 showed the most irregular fluctuation among the silicone oils. Initially, a release peak was observed, similar to the plot of control medium but starting at 1 week. A considerable drop in the quantifiable levels of the drug occurred at months 3 and 9, absolute drop in the dexamethasone released occurred at month 7 and 12.

All the quantified dexamethasone in samples acquired from silicone oil mediums converged at month 6, showing similar drug levels to the control medium at this point.

Quantitative values for DEX quantification in all samples are shown in Table 1.

**Table 1:** Dexamethasone quantification levels

Time	24 h	1 wk	2 wks	1 m	2 m	3 m	4 m	5 m	6 m	7 m	8 m	9 m	10 m	11 m	12 m
*BSS	6.89	8.55	7.8	1.61	8.73	8.8	8.57	8.66	8.16	8.42	9.26	9.25	9.28	9.3	8.88
*SO-1000	0	0	0	0	5.01	4.59	6.27	3.21	7.27	6.83	1.37	3.84	5.42	4.41	5.04
*SO-5000	0	0	3.55	0	3.5	4.18	5.41	5.68	7.34	5.98	4.39	0	1.3	0.98	3.14
*D-68	0	6.53	8.32	2.88	6.16	0.32	7.8	3.59	7.62	0	6.61	0.66	4.92	3.83	0

Table shows quantitative values of the dexamethasone released in samples obtained from different mediums: BSS, SO-1000, SO-5000 and D-68 at different times: 24 h, 1 week, 2 weeks and monthly for 12 months. ELISA test was performed to quantify the released drug and is reported in ng/mL

*BSS* basic saline solution, *SO-1000* 1000cS silicone oil, *SO-5000* 5000cS silicone oil, *D-68* heavy silicone oil, *ng/mL, *h* hours, *wk* week, *wks* weeks, *m* months

### Effect on TNF-α

TNF-α levels were analyzed to compare if released DEX from the implant had the same anti-inflammatory effect according to the medium it was placed.

**Fig. 4:**
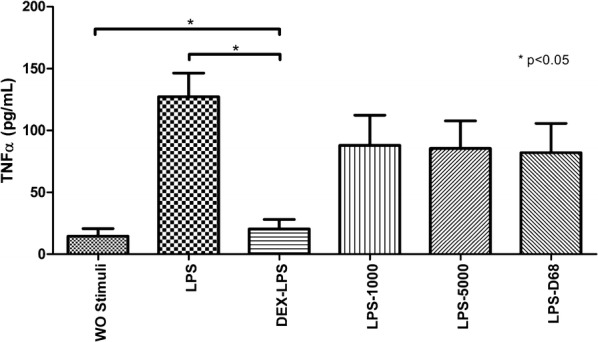
Inhibition model of dexamethasone and controls. A model of stimulation and inhibition was standardized using LPS-stimulated PBMNC (as a positive stimulation control) and DEX (as a positive inhibition control). PBMNC were also exposed to each of the three different kinds of SOs without DEX to assess if the SOs had any anti-inflammatory effect (inhibitory effect on TNF-α levels) by themselves. As a negative stimulation control, PBMNC were exposed to RPMI-1640 without any other stimuli. Results are presented in a bar graph where the mean value ± SD is reported in pg/mL. Mann–Whitney *U* test was used for statistical comparisons. A value of p < 0.05 was considered statistically significant. *BSS* basic saline solution, *LPS* lipopolysaccharide, *PBMNC* peripheral mononuclear blood cells, *SOs* silicone oils, *DEX* dexamethasone, *SD* standard deviation, *WO* without

A model of stimulation and inhibition was standardized (Fig. 4) where the LPS stimulated medium induced greater TNF-α concentrations (127.0 ± 33.66 pg/mL) compared to the negative stimulation control: 14.72 ± 10.46 pg/mL (p < 0.05).

The inhibition model where DEX was added showed a decrease in the production of TNF-*α* (20.6 ± 13.21 pg/mL) compared to the model stimulated only with LPS (127.0 ± 33.66 pg/mL) (p < 0.05).

The other controls were the different silicone oil mediums where any of the 3 silicones showed an inhibitory effect on TNF-α (LPS + SO-1000: 88.04 ± 41.87 pg/mL (p = 0.2); LPS + SO 5000 85.58 ± 38.35 pg/mL (p = 0.2); LPS + D-68: 82.01 ± 40.98 pg/mL (p = 0.4) in fact, the effects observed afterwards are not attributed to the presence of silicone oil but to the DEX released from the implant.

**Fig. 5:**
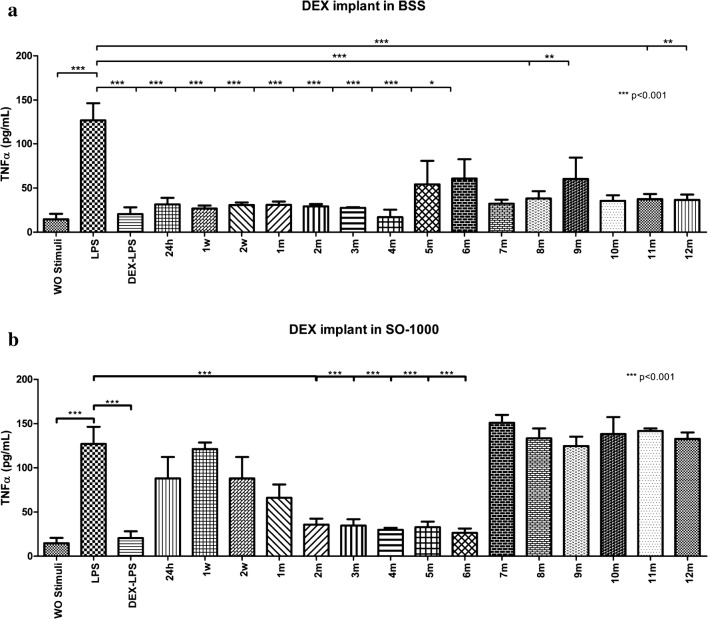
**a** Inhibitory effect on TNF-α levels from the implant in BSS. A model of stimulation and inhibition was standardized using LPS-stimulated PBMNC (as a positive stimulation control) and DEX (as a positive inhibition control). PBMNC were also exposed to each one of the samples acquired from the implant in BSS at different times to assess the inhibitory effects over TNF-α levels in every sample. As a negative stimulation control, PBMNC were exposed to RPMI-1640 without any other stimuli. Results are presented in a bar graph where the mean value ± SD is reported in pg/mL. Mann–Whitney *U* test was used for statistical comparisons. A value of p < 0.05 was considered statistically significant. *BSS* basic saline solution, *LPS* lipopolysaccharide, *PBMNC* peripheral mononuclear blood cells, *DEX* dexamethasone, *SD* standard deviation, *WO* without, *h* hours, *w* weeks, *m* months. *p < 0.05; **p < 0.01; ***p < 0.001. **b** Inhibitory effect on TNF-α levels from the implant in 1000cs SO. A model of stimulation and inhibition was standardized using LPS-stimulated PBMNC (as a positive stimulation control) and DEX (as a positive inhibition control). PBMNC were also exposed to each one of the samples acquired from the implant in SO-1000 at different times to assess the inhibitory effects over TNF-α levels in every sample. As a negative stimulation control, PBMNC were exposed to RPMI-1640 without any other stimuli. Results are presented in a bar graph where the mean value ± SD is reported in pg/mL. Mann–Whitney U test was used for statistical comparisons. A value of p < 0.05 was considered statistically significant. *SO-1000* 1000cS silicone oil, *LPS* lipopolysaccharide, *PBMNC* peripheral mononuclear blood cells, *DEX* dexamethasone, *SD* standard deviation, *WO* without, *h* hours, *w* weeks, *m* months. *p < 0.05; **p < 0.01; ***p < 0.001

Every sample of each medium at different times was analyzed. Showing that the DEX implant in the control medium (BSS) had inhibitory effects on TNF-α starting at 24 h (31.46 ± 12.68) (p < 0.001) and maintained during the 12-month period (Fig. 5a).

However, when the DEX implant is placed in 1000cS silicone oil, the inhibitory effect on TNF-α starts at month 2 (35.71 ± 11.82 pg/mL) (p < 0.001) and remains only until month 6 (26.38 ± 8.61 pg/mL) (p < 0.001) (Fig. 5b).

**Fig. 6:**
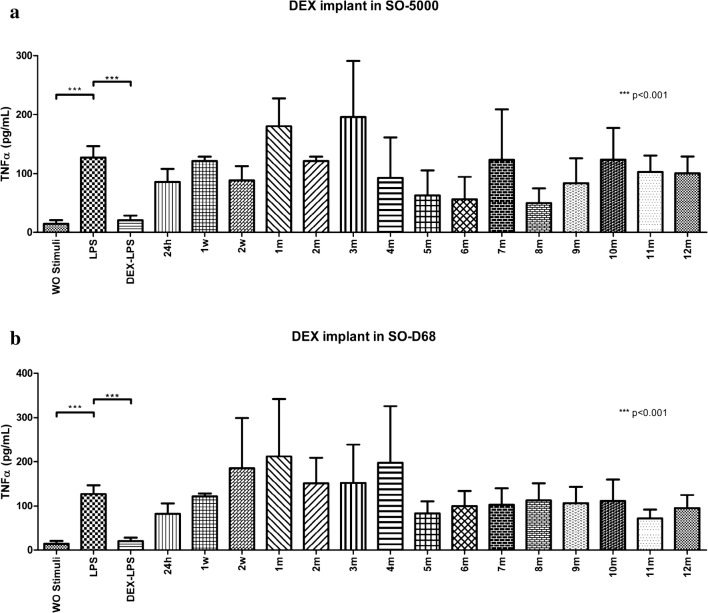
**a** Inhibitory effect on TNF-α levels from the implant in 5000cs SO. A model of stimulation and inhibition was standardized using LPS-stimulated PBMNC (as a positive stimulation control) and DEX (as a positive inhibition control). PBMNC were also exposed to each one of the samples acquired from the implant in SO-5000 at different times to assess the inhibitory effects over TNF-α levels in every sample. As a negative stimulation control, PBMNC were exposed to RPMI-1640 without any other stimuli. Results are presented in a bar graph where the mean value ± SD is reported in pg/mL. Mann–Whitney *U* test was used for statistical comparisons. A value of p < 0.05 was considered statistically significant. *SO-5000* 5000cS silicone oil, *LPS* lipopolysaccharide, *PBMNC* peripheral mononuclear blood cells, *DEX* dexamethasone, *SD* standard deviation, *WO* without, *h* hours, *w* weeks, *m* months. *p < 0.05; **p < 0.01; ***p < 0.001. **b** *Inhibitory effect on TNF*-*α levels from the implant in Heavy SO.* A model of stimulation and inhibition was standardized using LPS-stimulated PBMNC (as a positive stimulation control) and DEX (as a positive inhibition control). PBMNC were also exposed to each one of the samples acquired from the implant in D-68 at different times to assess the inhibitory effects over TNF-α levels in every sample. As a negative stimulation control, PBMNC were exposed to RPMI-1640 without any other stimuli. Results are presented in a bar graph where the mean value ± SD is reported in pg/mL. Mann–Whitney U test was used for statistical comparisons. A value of p < 0.05 was considered statistically significant. *D-68* heavy silicone oil, *LPS* lipopolysaccharide, *PBMNC* peripheral mononuclear blood cells, *DEX* dexamethasone, *SD* standard deviation, *WO* without, *h* hours, *w* weeks, *m* months. *p < 0.05; **p < 0.01; ***p < 0.001

DEX implants placed in 5000cS silicone oil and D-68 showed no inhibitory effect on TNF-α in any of the samples even when DEX concentrations were quantified (Fig. 6a, b)

## Discussion

According to the photographic follow-up, all the implants remained at the bottom of each test tube during the 12-month period and only the implant placed in BSS fully disintegrated starting at month 9 to be completely dissolved at 1 year. The implants inside the 3 silicone oils showed no changes in their form, migration or physical characteristics. Because of the in vitro design of this study, we could not replicate entirely the in vivo properties of the vitreous and the behavior of the DEX implant inside a human eye. One possible mechanism explaining our findings could be than in the human eye, constant movement and postural changes of the head might contribute to constant migration of the intravitreal implant in the presence of a pro-inflammatory enzyme rich environment at the posterior segment of the eye that contribute to the rapid disintegration of the implant, based on the device original patent [34] which states that release of the agent is achieved by erosion of the polymer followed by exposure of previously entrapped agent particles to the vitreous, with subsequent dissolution and release of the agent, and that the polymeric matrix will not be fully degraded until the drug load has been released. In our experimental model, the test tubes containing the implant remained static in a rack during the 12 months follow up. The only implant to achieve full disintegration at the end of the experiment (control medium) appeared as a turbid scattering of particles in the immediate surroundings of the tube’s bottom and even showed some sedimentation. This behavior can be explained simply by gravity the absolute lack of movement. A very interesting finding about the implants in silicone oils is that regardless of their densities, the implants remained unchanged at the bottom of the test tubes.

We found quantifiable DEX levels in all the samples of the control medium. Concentrations stabilized and remained constant since month 2 and correspond to previous descriptions in animal models by Chang-Lin et al. where the maximum concentrations of DEX were observed at 2 months after implant injection. The same authors already described similar pharmacokinetics of the DEX implant in vitrectomized rabbit eyes using an internal tamponade of BSS compared to non-vitrectomized eyes. The information available on the behavior of the DEX intravitreal implant in vitrectomized silicone oil-filled eyes is limited to some case reports where the implant remained trapped against the retina with no further analysis [32, 33]. Sherif and Wolfensberger [35] reported a retrospective case series of 5 patients with recurrent retinal detachments complicated by stage C PVR who underwent a pars plana vitrectomy with 5500cS SO tamponade and adjunctive intravitreal injection of a 0.7 mg DEX implant. The authors concluded that the DEX implant is well tolerated in conjunction with SO tamponades in eyes with retinal detachment and PVR, based on their anatomic outcomes and the lack of adverse events found in their study. However, even when this results might represent a closer idea of what to expect when a DEX implant is injected in a vitrectomized silicone oil-filled eye, it fails to answer if there was any release of the drug from the implant or if it ever reached therapeutic concentrations. Because samples were not acquired from the silicone oil-filled vitreous cavity, the answer to this questions may remain a matter of speculation.

Figure 3 shows quantification of DEX released from implants in each medium at different times. An irregular plot representing the concentrations of the silicone oil mediums is evident due to some samples that showed zero or almost zero levels of DEX quantification and the subsequent samples showed levels as high as the control medium. We attributed this behavior to the method to acquire the samples from the test tubes. We measured the volume of each tamponade medium inside the test tube and acquired the 200 µL sample from the center, fairly away from the implant that remained at the bottom of the test tube. We assume that in the denser mediums, the DEX release is limited to the immediate surrounds of the implant or it remains against the bottom of the test tube, and in the less dense mediums such as BSS and 1000cS silicone oil, the released DEX reached the pipette as the drug could have been more evenly distributed among the tamponade medium. Analyzing this situation, we believe that if the test tubes had been tilted of moved at a determined time during the experiment to simulate the constant movement of a human eye, maybe the drug released from the implants could had been homogeneously distributed in the total volume of the test tubes to reach the pipette and the quantification of DEX in the samples would show a more regular behavior.

Another interesting finding about this experiment is the fact that among the 3 different densities of silicone oil studied, only SO-1000cS showed an anti-inflammatory effect over TNF-α but the effect lasted only for 4 months (from month 2 to 6) even when the bar graph in Fig. 5b shows a downward trend starting from the bar at 2 weeks and this can be assumed as inhibitory effects, this interpretation contradicts with the quantitative values displayed in Table 1 because there were absolute zero concentrations of DEX released before month 2. Also, this supposed inhibitory effect observed in absence of quantifiable drug release must not be attributed to the anti-inflammatory effect of silicone oils according to our model of stimulation/inhibition where we could not demonstrate any inhibitory effects using only SO, which means that SO do not have an anti-inflammatory effect by themselves.

A similar finding was observed in the denser SO (SO-5000 and D-68) where Fig. 6a, b show a graph where some of the bars indicate an inhibitory effect, however, quantitative values in some of these samples are again quantified at zero levels.

A possible explanation to this situation may be the quantification method. Debatable opinions may arise when considering that a very viscous solution such as the silicone oils samples were analyzed by ELISA test arguing that this might have altered the testing results. However, the authors performed multiple previous ELISA tests where a volume of silicone oil was mixed with an amount of a known protein achieving 100% of recovery. Nevertheless, this does not fully explain the findings previously described.

## Experiment considerations and limitations

As we previously stated, the in vitro nature of this study limits us to exactly replicate the vitreoretinal pharmacokinetics of the implant in the human vitreous; however the aim of this study was to determine the behavior of the DEX implant in silicone oil. When this experiment was designed, the most questionable variable considered by the authors was the fact that in a vitrectomized silicone-oil filled eye, regardless of the viscosity of the SO used as tamponade, there is always a layer of aqueous surrounding the SO as part of the physiological and constant production of aqueous that eventually reaches the posterior segment of the eye. Considering this, before the DEX implant injection in each test tube, we created some previous similar experimental models adding a layer of BSS inside the test tubes with silicone oil to simulate the aqueous meniscus formed in a SO-filled eye. We encountered several problems with this model. First, our experiment included silicone oils with different densities, SO-1000cS and SO-5000cS are both lighter-than-water silicones, which means that the layer of BSS remained at the bottom of the tube and probably if a DEX implant was injected to this test tube, it would remain at the bottom of the tube and would only be bathed in BSS. In the D-68 test tube, the BSS layer remained above the SO due to its heavier-than-water properties, this would mean that if a DEX implant was injected inside this test tube it would remain at the bottom and it would be bathed only in SO. Also, acquiring samples from the DEX implant in D-68 would mean that the pipette would always have to break through the BSS layer to reach for the SO layer underneath and this would be likely to alter the results because the other SOs samples would never be in contact with BSS. Hence, the authors intentionally omitted adding a layer of BSS to the SO test tubes.

One considerable limitation of this study was the fact that the test tubes remained static. Adding constant movement would have definitely impacted the results. Future experiments can be performed based on our current model, adding a BSS layer to the SOs test tubes and keep them in constant movement to simulate better the expected behavior of the DEX implant in the human eye.

## Conclusions

In summary, denser tamponade mediums are likely to alter the pharmacokinetics of the DEX intravitreal implant. Our results suggest that the intravitreal DEX implant in dense silicone oil-filled eyes should be avoided, due to different pharmacokinetics observed.

Even with the limitations of this study, the results of the experiment set a pattern of what should be expected from the use of the DEX intravitreal implant in silicone oil.
